# Relation between Dopamine Synthesis Capacity and Cell-Level Structure in Human Striatum: A Multi-Modal Study with Positron Emission Tomography and Diffusion Tensor Imaging

**DOI:** 10.1371/journal.pone.0087886

**Published:** 2014-01-31

**Authors:** Hiroshi Kawaguchi, Takayuki Obata, Harumasa Takano, Tsuyoshi Nogami, Tetsuya Suhara, Hiroshi Ito

**Affiliations:** 1 Molecular Imaging Center, National Institute of Radiological Sciences, Chiba, Japan; 2 Research Center for Charged Particle Therapy, National Institute of Radiological Sciences, Chiba, Japan; University of Manchester, United Kingdom

## Abstract

Positron emission tomography (PET) study has shown that dopamine synthesis capacity varied among healthy individuals. This interindividual difference might be due to a difference in the cell-level structure of presynaptic dopaminergic neurons, i.e., cellular density and/or number. In this study, the relations between the dopamine synthesis capacity measured by PET and the parameter estimates in diffusion tensor imaging (DTI) in striatal subregions were investigated in healthy human subjects. DTI and PET studies with carbon-11 labeled L-DOPA were performed in ten healthy subjects. Age-related changes in the above parameters were also considered. Fractional anisotropy showed a significant positive correlation with age in the posterior caudate. There was significant negative correlation between dopamine synthesis capacity and mean diffusivity in the posterior caudate and putamen. Assuming that mean diffusivity reflects the density of wide-spreading axonal terminals in the striatum, the result suggests that dopamine synthesis may be related to the density of dopaminergic neuronal fibers. It is evident that PET/DTI combined measurements can contribute to investigations of the pathophysiology of neuropsychiatric diseases involving malfunction of dopaminergic neurons.

## Introduction

The central dopaminergic system is of great interest in the pathophysiology of neuropsychiatric diseases such as Parkinson's disease and schizophrenia. To assess the capacity of endogenous dopamine synthesis, one of the presynaptic dopaminergic functions, carbon-11 labeled L-DOPA (L-[β-^11^C]DOPA), is used as radioactive tracer for positron emission tomography (PET) [1]–[3]. The relative activity of cerebral aromatic L-amino acid decarboxylase (AADC) representing endogenous dopamine synthesis capacity can be measured by L-[β-^11^C]DOPA [4], [5]. A PET study has reported that dopamine synthesis capacity varied among healthy individuals [2]. Random noise during PET measurement could be one of the reasons for this variation. In addition, genotypes of human monoamine-synthesizing enzymes, e.g., tyrosine hydroxylase (TH) and AADC, have been determined [6], and it was reported that the endogenous dopamine synthesis capacity measured by PET was correlated with personality traits [7]. These findings indicate that the variations in dopamine synthesis capacity could be related to the physiological interindividual differences in presynaptic dopaminergic functions.

One of the possible reasons for the interindividual differences in presynaptic dopaminergic functions could be the difference in the cell-level structure of presynaptic dopaminergic neurons, i.e., cellular density and/or number. Actually, the density of tyrosine hydroxylase cells in autopsied substantia nigra correlates with striatal dopamine levels in premortem positron emission tomography with 6-[^18^F] fluoro-L-DOPA ([^18^F]FDOPA) [8]. Also, an autoradiography study of rats showed statistically significant correlation between the left side/right side [^18^F]FDOPA uptake ratio and number of nigral dopaminergic cells [9]. It has been widely considered that the parameter estimates in diffusion tensor imaging (DTI), which measures restricted water diffusion in biological tissue ([10]), could reflect the cell-level structure in tissue, including cellular density and/or number. Thus, the interindividual variation of dopamine synthesis capacity measured by PET could be related to that of DTI parameter estimates. The human striatum can be divided into 5 anatomic subregions, i.e., the ventral striatum and the anterior/posterior putamen and anterior/posterior caudate [11]. Subregional features of the striatum were first reported in a postmortem study [12], and subsequently in dopaminergic PET studies [13], [14] and DTI studies [15], [16] in vivo. These results suggest that subregional dependency may affect the relation between dopamine synthesis and DTI metrics.

In this study, the relations between dopamine synthesis capacity and the parameter estimates in DTI in striatal subregions were investigated in healthy human subjects. PET with L-[β-^11^C]DOPA and magnetic resonance imaging (MRI) were performed in the same subjects. The relations between parameter estimates and age were also analyzed because age-related decreases in cellular number can affect water diffusion. To the best of our knowledge, this study is the first to discuss the relationship between dopamine synthesis capacity and cellular density and/or number in humans in vivo.

## Materials and Methods

### 2.1 Ethics Statement

This study was approved by the Ethics and Radiation Safety Committees of the National Institute of Radiological Sciences, Chiba, Japan. All participants gave their written informed consent.

### 2.2 Data acquisition

Ten healthy volunteers, (8 males and 2 females, 22 to 67 years old, 42.6±17.0, mean ± SD), participated in the study. The subjects were free of somatic, neurological or psychiatric disorders on the basis of their medical history and MRI of the brain. PET scans were performed using an ECAT EXACT HR+ system (CTI-Siemens, Knoxville, TN, USA) in three-dimensional mode, which provides 63 planes and a 15.5-cm field-of-view (pixel size 2.68×2.68×2.42 mm^3^, in-plane matrix 128×128). L-[β-^11^C]DOPA was synthesized from [^11^C] carbon dioxide via D,L-[3-^11^C]alanine as described previously [17], [18]. Kinetic analysis with L-[β-^11^C]DOPA is less affected by 3-O-methyl metabolites than that with [^18^F]FDOPA, a well-known radio tracer for assessing dopamine synthesis capacity [1], [19]. After a 10-min transmission scan with a ^68^Ge–^68^Ga source, a bolus of 258–392 MBq of L-[β-^11^C]DOPA was injected with specific radioactivity of 12.2 GBq/µmol to 81.1 GBq/µmol at the time of injection into the antecubital vein with a 20-ml saline flush. Dynamic PET scanning was started simultaneously with the tracer injection and continued for 64 min, consisting of seven 1-min frames, five 2-min frames, four 3-min frames, and seven 5-min frames [20]. Scatter was corrected by a single scatter simulation technique [21]. All emission scans were reconstructed with a Hanning filter with a cut-off frequency of 0.4 (full width at half maximum = 7.5 mm).

MR images were acquired using a Philips Intera 1.5 tesla MR unit (Philips Medical Systems, Best, The Netherlands) on the same day as the PET scan. Scan parameters for T1-weighted (T1W) image acquisition were 1-mm thick 3D T1W images with a transverse plane (repetition time, TR/echo time, TE = 21/9.2 ms, flip angle = 30°, matrix = 256×256, field-of-view = 256×256 mm), yielding 196 contiguous brain slices. Diffusion-weighted images were acquired by single-shot echo-planar imaging with the sensitivity-encoding (SENSE) parallel-imaging scheme (reduction factor = 2.0, TR = 8645 ms, TE = 96 ms). The field-of-view was 240×240 mm^2^ (nominal resolution, 2.5 mm), with an imaging matrix of 96×96 zero-filled to 256×256 pixels. Sixty continuous transverse slices were acquired with 2.5-mm slice thickness, and b-values of 0 s/mm^2^ and 700 s/mm^2^. The diffusion gradient pulse duration and separation were δ = 24 ms and Δ = 24 ms, respectively. Diffusion was measured along six non-collinear directions: (x, y, z) = [(1, 0, 0), (0, 1, 0), (0, 0, 1), (−0.707, 0.707,0), (0.707, 0, −0.707), (0, 0.707, −0.707)]. Acquisition was repeated 30 times to enhance the signal-to-noise ratio, and the total scan time was 40 min.

### 2.3 Data analysis

The striatum has sufficient contrast with surrounding tissue (white matter and cerebral spinal fluid) in T1W images. Thus, regions of interest (ROIs) of the striatum were defined in bilateral hemispheres on the individual T1W images. Thereafter, morphological erosion was applied to the ROIs, as the selected ROI may have been affected by the partial-volume effect, especially at the edge pixels. Subregional boundaries in the striatum, i.e., anterior and posterior caudate nuclei (ACN and PCN), anterior and posterior putamen (APT and PPT) and nucleus accumbens (NA), were defined on the ROIs according to the previous report (Martinez et al., 2003). An ROI of the occipital cortex (OC) was also defined on the T1W image and used as reference region to calculate dopamine synthesis capacity, as there is only a little irreversible binding in this region.

The DTI parameters were estimated with FMRIB's Diffusion Toolbox (FDT; version 2.0) in the FMRIB Software Library (FSL, version 4.1.8; Oxford Centre for Functional MRI of the Brain, Oxford, UK). Each directional volume from the diffusion data set was resampled to the b = 0 image to correct for the remaining eddy current distortion as well as to correct for participant motion due to the long acquisition time. Mean diffusivity (MD) and fractional anisotropy (FA) were calculated and used in the analysis.

The PET and DTI images were spatially co-registered to the T1W images by using SPM 8 (Wellcome Trust Centre for Neuroimaging, London, UK). Non-diffusion weighted (b = 0) image and summation of PET frames were used to estimate the transformation matrices. Time activity curves (TACs) were obtained by averaging the radioactivity in the bilateral subregional ROI of each time frame. A ratio method was applied to calculate the dopamine synthesis capacity by enhancing the signal-to-noise ratio [3]. There are linear relations between the dopamine synthesis capacity obtained by the ratio method, the graphical method and the conventional arterial blood sampling approach [1], [22]. The dopamine synthesis capacity, R, was calculated by the following equation [22]:
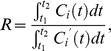
where the integration interval (t_1_, t_2_) is (29, 64) minutes, and *C*_i_ and *C*_i_′ are the TACs of the striatal subregions and OC, respectively. The diffusion estimates in subregional ROIs were also calculated by averaging each bilateral ROI on the T1W image space.

All statistical tests were performed by functions in the statistical toolbox of MATLAB (MathWorks, Natick, MA, USA). The subregional differences were analyzed by Friedman's test and post-hoc multi-comparisons with Bonfferoni correction. The relations between R and DTI estimates were evaluated by Spearman's correlation coefficients with and without controlling for age. The relations between age and DTI estimates were also evaluated because of the wide age range of the subjects. P-values less than 0.05 were considered statistically significant.

## Results

**Figure 1:**
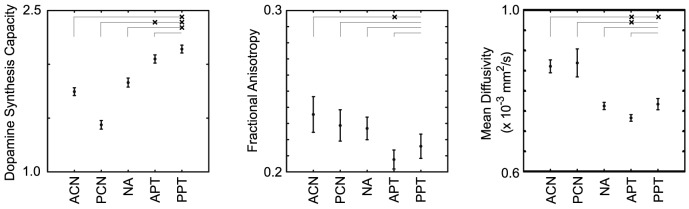
Subregional differences in dopamine synthesis capacity, fractional anisotropy and mean diffusivity in the striatum evaluated by Friedman's test (α = 0.05). The cross (x) represents statistically significant difference between two regions. Error bar shows standard error of means.

The subregional DTI estimates and dopamine synthesis capacity R were represented in Table 1 and the differences evaluated by Friedman's test are shown in Fig. 1 (α = 0.05). Each estimate showed subregional differences. Post-hoc analysis showed statistically significant differences between fractional anisotropy (FA) of APT and ACN. Mean diffusivity (MD) showed significant differences between PCN-APT, ACN-PPT and PCN-PPT pairs. R showed significant differences between ACN-PPT, PCN-APT, ACN-PPT and NA-PPT pairs.

**Table 1:** Average and standard error of mean of dopamine synthesis capacity, fractional anisotropy and mean diffusivity in striatal subregions.

	ACN	PCN	NA	APT	PPT
R	1.746±0.036	1.438±0.040	1.831±0.042	2.050±0.038	2.141±0.036
FA	0.236±0.011	0.229±0.010	0.227±0.007	0.208±0.006	0.216±0.008
MD	0.859±0.160	0.868±0.035	0.761±0.009	0.731±0.008	0.766±0.014

ACN: Anterior caudate nucleus, PCN: posterior caudate nucleus, NA: nucleus accumbens, APT: anterior putamen, PPT: posterior putamen, R: dopamine synthesis capacity, FA: fractional anisotropy, MD: Mean Diffusivity (×10^−3^ mm^2^/s).

The age-related changes of DTI estimates and R were evaluated by Spearman's correlation coefficient. FA showed a significant positive correlation (ρ = 0.717, p = 0.020) with age in PCN. The other estimates did not show any age-related correlation.

**Figure 2:**
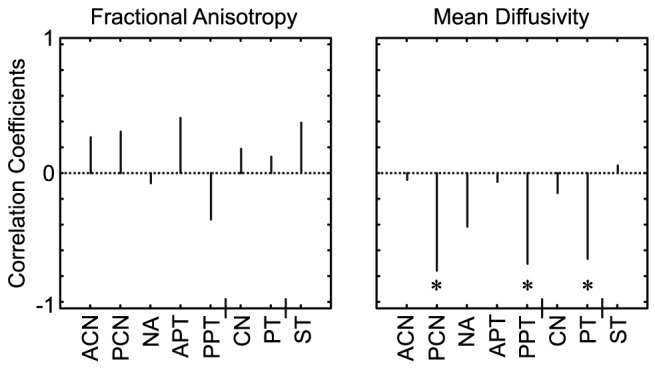
Age-adjusted Spearman's correlation coefficient of dopamine synthesis capacity versus fractional anisotropy and mean diffusivity in striatal subregions. Asterisks (*) represent statistical significance (P<0.05).

**Figure 3:**
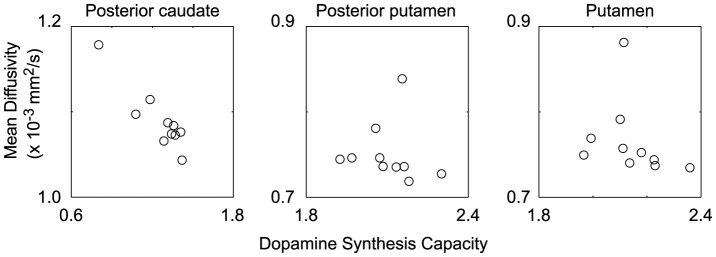
Scatter plots of dopamine synthesis capacity versus mean diffusivity in posterior caudate and posterior and whole putamen.

**Figure 4:**
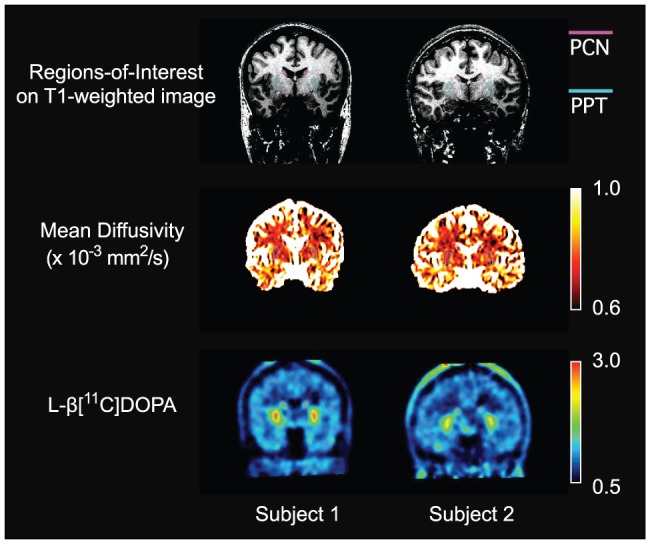
Typical images for subjects with high and low mean diffusivity (subjects 1 and 2, respectively) and corresponding mean diffusivity images. Regions of interest on T1-weighted images for posterior caudate and putamen (PCN and PPT) are also shown.

The relations between R and DTI parameter estimates were evaluated by Spearman's correlation coefficient and summarized in Fig. 2. There was no statistical correlation between FA and R. The partial correlation coefficient controlling for age showed significant negative correlations between R and MD in PCN (ρ = −0.758, p = 0.018), PPT (ρ = −0.685, p = 0.035) and PT (ρ = −0.667, p = 0.050). Scatter plots of R versus MD for PCN, PPT and PT are shown in Fig. 3. Typical images for subjects with high and low R (subjects 1 and 2, respectively) and corresponding MD images are shown in Fig. 4, together with ROIs on T1W images.

## Discussion

There were statistically significant differences in dopamine synthesis capacity among striatal subregions. A previous postmortem report showed the distribution of dopamine transporter in the striatum to differ, based on the regional dependencies of the dopamine D1, D2 and D3 receptors [23]. There are several reports suggesting the relation of subregional dopaminergic function in the striatum with psychiatric disorders [24] and with cognitive function [25]. As with distributions of dopamine transporter and receptors, distribution of dopamine synthesis capacity might be relevant to the interpretation of neurological function and psychiatric disorders. The difference of diffusion metrics among subregions may reflect cellular-level structural difference of these functionally different cells. Actually, immunohistological study on postmortem human brain has demonstrated that the architecture of the human striatum in terms of its interneuron composition varies in functional territories [26].

FA had a significant positive correlation with age in PCN in this study. The other regions, while not statistically significant, also showed positive correlations. Similar results were also reported in a number of other studies ([27]–[32]. The precise etiology for these differences is unknown, but some speculations were put forth: relation of local iron content [31], [32], losses of isotropic cell structures and pervasive axons [27], and change in cellularity caused by diseases such as Huntington's disease, cerebral hypertension and cerebral edema [28]. In addition to the above hypotheses, the loss of randomly spreading terminals of dopaminergic neurons originating in the substantia nigra might be a possible reason for the age-related increase in FA. In any case, a microphysiological explanation is required to confirm the above speculations.

The physiological meanings of MD have yet to be established. However, it has been considered that MD mainly reflects the intra- and extra-cellular water motion restricted by cell membranes [33], [34]. This means that, as cell density in tissue is greater, water mobility decreases, resulting in a decrease in MD. The main cell-type in the striatum is the medium spiny GABAergic neuron [35]. These neurons send axons to the internal and external segments of the globus pallidus as well as to the substantia nigra pars reticulata. The density of GABAergic neurons can affect MD in the striatum because they account for a large amount of the striatal neuronal volume [26]. Another source of the interindividual variation of MD might be the wide-spreading axonal terminal distribution from the substantia nigra. TH-positive dopaminergic neurons possess widely-spread and highly-dense axonal arborizations in the neostriatum of rats in the study of Matsuda et al. [36]. They showed that the striatal axonal bush of each dopaminergic neuron covered 0.45–5.7% of the total volume of the neostriatum. This indicates that the striatal density of dopaminergic axons can affect MD in the striatum.

An immune-histological study with rats demonstrated that dopamine is distributed widely throughout the whole striatum, as are TH and AADC [37]. R depends on the activity of AADC, as it is the enzyme that converts DOPA to dopamine, which is then stored in vesicles of dopaminergic neurons in the striatum [38]. AADC mainly locates in the caudate and putamen in human brain [39]. The location candidates of AADC in the striatum are dopaminergic fibers originating from neurons in the substantia nigra, serotonergic fibers arising from the caudal brainstem, and non-catecholaminergic AADC neurons that presumably exist in the striatum [40]. Although serotonergic fibers and non-catecholaminergic neurons are not negligible, AADC will mainly locate in dopaminergic neuron terminals projected from the substantia nigra to the striatum. Actually, the degree of cell loss in the substantia nigra strongly correlates with the dopamine concentration in the putamen and caudate [41]. Thus, one of the major sources for the interindividual variation of R may be the amount of AADC in the dopaminergic neuron terminal.

As mentioned in the above two paragraphs, the striatal axonal bush of dopaminergic neurons affects either R or MD values. The results showed a negative correlation between R and MD in PCN, PPT and PT, suggesting that a higher density of the axonal bush can represent the lower MD and higher R, and a lower density can represent lower R and higher MD. Further studies such as microscopic observations and/or multi-modal imaging with DTI and other PET tracers for measurement of dopaminergic presynaptic functions, e.g., dopamine transporter, are necessary to arrive at a solid conclusion. In addition, previous reports have shown a negative correlation between presynaptic dopamine synthesis capacity and dopamine D_2_ receptor binding in healthy volunteers [42]. Thus, MD may also be related to dopamine D_2_ receptor binding.

The present study has the limitation that the findings are based on a small number of subjects. Post-hoc statistical power analysis demonstrated that statistical power (1-β, usually 0.8 is recommended) for 0.758, 0.685 and 0.667 of correlation coefficients was 0.794, 0.649 and 0.613, respectively, for the hypothesis that the correlation between R and MD is 0 in 10 subjects. Thus, the present results have a little higher second type of statistical error. Actually, we could not find an age-related decline of dopamine synthesis in ST, as previously reported [3]. In addition, the physiological background of DTI metrics in gray matter is still controversial. However, there is no question that these metrics reflect water motions restricted by the cell-level structure of tissue. The new diffusion MRI, such as Q-space imaging and diffusion kurtosis imaging, with a high magnetic field scanner may provide more detailed information. The insufficient spatial resolution of PET is also a critical issue in respect to the reliability of the present results. Actually, R of PCN ROI is lower than that of the others due to the thin-tube-like shape. However, specific binding of radiotracers is not observed in tissues surrounding the ST, which means that the PET pixel intensity of the PCN region is not contaminated by radioactivity of surrounding tissues. The PET and DTI scanner with higher spatial resolution and sensitivity will resolve this issue as well as enable us to perform more detailed analyses of the central dopaminergic system, e.g., analysis of PET/DTI relations on nigrostriatal pathways and cerebral cortical regions including the limbic system by measuring the small substantia nigra and cerebral cortex expressing small amounts of AADC.

## Conclusions

We found a negative relation between R and MD in PCN and PPT. Assuming that water motion is related to cellularity, dopamine synthesis may depend on the density of dopaminergic neuronal fibers. PET/DTI combined measurements can be expected to contribute to the investigation of neuropsychiatric diseases involving malfunction of dopaminergic neurons such as in Parkinson's disease, and will play a major role with the increasing availability of integrated PET/MRI scanners.
